# BK viremia in critically ill surgical patients with hemorrhagic or septic shock

**DOI:** 10.1186/1756-0500-5-100

**Published:** 2012-02-16

**Authors:** Maximilian Nass, Benedikt Weissbrich, Moritz Huber, Elisabeth Marion Schneider, Manfred Weiss

**Affiliations:** 1 Department of Anaesthesiology, University Hospital Ulm, Steinhoevelstr. 9, 89075 Ulm, Germany; 2 Institute of Virology and Immunobiology, University Wuerzburg, Versbacher Str.7, 97078 Wuerzburg, Germany; 3 Department of Experimental Anaesthesiology, University Hospital Medical School Ulm, Steinhoevelstr. 9, 89075 Ulm, Germany

**Keywords:** Acute kidney injury, Critically ill, Polyomavirus, BK virus, Sepsis, Shock

## Abstract

**Background:**

Infections with polyomavirus BK virus (BKV) are a common cause of renal dysfunction after renal transplantation and may also be harmful in surgical patients with shock. The aim of the present study was to determine the frequency of BKV viremia in critically ill surgical patients with septic or hemorrhagic shock, and, if viremia is detectable, whether viremia may be associated with renal dysfunction.

**Findings:**

A total of 125 plasma samples from 44 critically ill surgical patients with septic or hemorrhagic shock were tested by real-time polymerase chain reaction (PCR) for BKV DNA during their stay on the intensive care unit (ICU). BKV viremia occurred in four patients, i.e. in three of the septic and in one of the hemorrhagic shock group. There was no association between viremia and renal dysfunction. All positive samples contained a low viral load (< 500 copies/ml).

**Conclusions:**

Since BK viremia was rarely found and with low viral load only in critically ill surgical patients with shock, it is very unlikely that BK viremia results in BK nephropathy later on.

## Background

Infections with BK virus (BKV) are an increasing problem and a common cause of renal dysfunction after allograft renal transplantation [1,2]. IgG seroprevalence for BK virus (BKV) was 82% in healthy blood donors, and, over a period of 14 months, no BKV viruria was detectable [3]. Kaneko and colleagues reported prevalence of BKV in urine samples in about 13.5% of the healthy control group, 33.3% of patients with chronic renal disease, and 55.6% of patients with renal disease under cortisone therapy [4].

Critically ill nonimmunosuppressed patients with either trauma or sepsis have been reported to demonstrate immunologic features consistent with immunosuppression ("immunoparalysis"), i.e. cytomegalovirus or herpes simplex virus reactivation or infections [5-8]. Thus, we hypothesized that BKV viremia might also occur in critically ill surgical patients. Since the appearance of a BKV nephropathy was strongly associated with the viral load detected in renal transplant patient's blood [1], we focused on detection of viral load in the blood of surgical critically ill patients. The aim of the present study was to determine the frequency of BKV viremia in surgical critically ill patients with hemorrhagic or septic shock.

## Methods

### Patients and data collection

The prospective study is in compliance with the Helsinki declaration and was approved by the Independent Ethics Committee of the University Ulm (65/07). All surgical patients admitted to the Anaesthesiology adult ICU between August 2008 and December 2008 with severe SIRS/sepsis, septic or haemorrhagic shock on admission or developing septic or haemorrhagic shock on the ICU were included in the present study, in total, 51 cases ≥ 18 years with shock. Four patients of the hypovolaemic shock group and three patients of the septic shock group had to be excluded from further analysis, due to refusal of informed consent, stay < 24 hours on ICU, early demission to another ICU or concomitant cardiogenic shock. Thus, in total, blood samples of 44 critically ill shock patients were included for analysis of BKV deoxyribonucleic acid (DNA), 18 in the septic shock group and 26 in the hemorrhagic shock group (http://ClinicalTrials.gov ID: NCT00736827). Blood samples were taken before, during and after shock: on the first days of severe SIRS/sepsis; shock with a noradrenaline dose in μg/kg × min of > 0 and ≤ 0.1 > 2 h, > 0.1 and ≤ 1.0 > 2 h, > 1.0 > 2 h, or > 1.0 + adrenaline > 2 h; after septic shock; before demission from the ICU or before death.

### Diagnosis of sepsis, acute kidney injury (AKI) and severity of organ dysfunctions and of disease

Sepsis and septic shock were defined using the 2003 SCCM/ESICM/ACCP/ATS/SIS sepsis definitions [9]. Shock was defined as hypotension despite adequate volume resuscitation, a systolic blood pressure of ≤ 90 mmHg, or the need of vasopressors to keep blood pressure ≥ 90 mmHg.

Acute kidney injury (AKI) was defined by the Acute Kidney Injury Network criteria [10], which are based on acute alterations in serum creatinine or urine output. Severity of organ dysfunctions was evaluated daily by the SOFA score (Sequential Organ Failure Assessment) [11], and of sepsis by the 2003 sepsis definitions [9].

Severity of disease on admission was monitored by the SAPS 3 score (Simplified Acute Physiology Score 3) [12] and the APACHE II score (Acute Physiology and Chronic Health Evaluation II) [13].

### Blood samples

Blood samples were taken from arterial lines of the patients at the ICU. Samples were immediately centrifuged and plasma was stored at -20°C until testing.

### Detection of BKV DNA by real-time PCR

The presence of BKV DNA was tested via real-time polymerase chain reaction (PCR) [14]. DNA was extracted from 200 μl of the plasma samples using the High Pure viral nucleic acid kit (Roche, Mannheim, Germany) according to the instructions of the manufacturer. The efficiency of the nucleic acid purification was controlled by co-extraction and subsequent amplification of Herpes virus saimiri DNA. The BKV real-time PCR was performed in a final volume of 20 μl consisting of 5 μl of DNA, primers, probe at a final concentration of 200 nM each, and 1 × Quantitect probe master mix (QIAGEN, Hilden, Germany). For real-time BK PCR, primer 1 with sequence (5'-3')AGCAGGCAAGGGTTCTATTACTAAAT, primer 2 with GAAGCAACAGCAGATTCTCAACA, and probe with AAGACCCTAAAGACTTTCCCTCTGATCTACACCAGTTT were used [14]. Amplification was performed on a 7500 real-time PCR system (Applied Biosystems, Darmstadt, Germany). The cycling conditions were 50 cycles with 30 s at 95°C and 60 s at 60°C after a preheating step of 15 min at 95°C. The lower limit of quantification was 300 copies/ml. General laboratory procedures to prevent PCR contamination were strictly adhered to. One negative probe was extracted and amplified for every five samples.

### Statistical analyses

Rates of BKV DNA detection in blood of consecutive patients are given. No statistical analyses were performed.

## Results

The study included 44 patients, 18 in septic shock and 26 in hemorrhagic shock.

Patient's characteristics and scores are summarized in Table 1. Median total stay in the ICU was 9 days (range 2-52) and in the hospital 30 days (range 3-117), respectively. Twenty-two patients had no acute kidney injury (AKI), and 22 AKI stages 1 to 3. 9/44 patients required renal replacement therapy (RRT).

**Table 1:** Patient characteristics and scores

		Total	Shock		RRT		Maximal acute kidney injury stage			
			**Hemor-rhagic**	**Septic**	**Yes**	**No**	**Stage 0**	**Stage 1**	**Stage 2**	**Stage 3**

Patients	n =	44	26	18	9	35	22	13	6	3

Age, years	Median Range	56.5	52.5	60	69	53	54.5	51	68.5	63
		20-88	20-81	37-88	45-73	20-88	21-88	20-81	60-71	45-70

Male/female	n =	35/9	19/7	16/2	8/1	27/8	16/6	11/2	5/1	3/0

Stay in hospital before ICU, days	Median	1	1	4	3	1	1	0	5	2
		
	Range	0-36	0-14	0-36	0-15	0-36	0-36	0-19	2-15	2-5

Days on ICU	Median	9	8	12	17	7	6	15	17	25
		
	Range	2-52	3-52	2-34	3-43	2-52	2-24	3-28	7-52	5-34

Stay in hospital after ICU	Median	12	9	17	5	15	12	11	17	11
		
	Range	0-111	0-48	0-111	0-41	0-111	0-111	0-46	0-41	11-25

Days in hospital	Median	30	24.5	44	24	31	24	29	54	50
		
	Range	3-117	3-73	3-117	3-73	5-117	3-117	3-65	20-73	18-52

Period under BKV observation, days before + on ICU	Median	14	10	19.5	20	13	7.5	16	26.5	27
		
	Range	3-57	3-57	3-43	3-57	3-54	3-43	3-35	9-57	7-39

SOFA score	Median	9	8	9	13	8	7.5	9	9.5	12
		
	Range	3-18	3-15	5-18	9-18	3-12	4-12	3-18	7-15	10-16

SAPS3 score	Median	45	47	42.5	66	45	45	45	56.5	45
		
	Range	24-87	26-87	24-87	26-87	24-71	33-71	24-87	39-87	26-68

APACHE II score	Median	20	22.5	18	30	20	21	18	24.5	18
		
	Range	10-45	10-45	11-37	16-45	10-36	11-30	10-37	15-45	16-33

ICU survival/death		39/5	24/2	15/3	6/3	33/2	21/1	10/3	5/1	3/0

RRT/no RRT		9/35	3/23	6/12	/	/	1/21	3/10	3/3	2/1

Total days with AKI	Median	1	0	1	7	0	0	1	8	20
		
	Range	0-26	0-13	0-26	0-26	0-8	0	1-9	2-13	4-26
	
AKI on demission from hospital	n =	13	4	9	5	8	2	7	3	1

BKV reactivation	n =	4	1	3	0	4	3	0	1	0

*AKI *acute kidney injury; *RRT *renal replacement therapy; *SOFA *highest value during ICU stay; SAPS3 and APACHE II: value on admission to ICU

At least two, and up to five blood samples were collected per patient. A total of 125 plasma samples was analyzed for BKV DNA. Results regarding viral load before, during and after shock are given in Table 2. In total, five blood samples of four patients were tested positive for BKV DNA. The viral load in all positive samples was low (≤4.4 × 10^2 ^copies/ml). One septic shock patient, only, revealed AKI stage 2 at maximum and BKV viremia. None of the four BKV positive patients needed RRT (Tables 1 and 2). None of the nine patients with RRT was tested positive for BKV viral DNA.

**Table 2:** BKV reactivation in patients with hemorrhagic or septic shock

**Pat. no**.	Shock group	Days in hospital before ICU	Days on ICU	Days in hospital after ICU	Total days in hospital	Day at ICU with BKV detection			Viral load (copies/ml)		Survival	AKI 1 days	AKI 2 days	AKI 3 days	AKI stage on demission
												
							1st day severe SIRS/sepsis	1st day shock	1st day after shock	before demission/death					
37	hemorrhagic	0	11	4	15	4			< 300*		+	0	0	0	0

5	septic	0	6	111	117	2	440				+	0	0	0	0

19	septic	2	4	0	6	2 and 4		< 300		< 300	-	0	0	0	0

29	septic	6	15	25	46	3			< 300		+	6	2	0	2

*300: BKV DNA weakly positive below the limit of quantification

## Discussion

The main result of the present study is that BKV viremia was rarely found in critically ill surgical patients with shock and with low viral load, only.

Due to the small sample size, conclusions have to be drawn with caution. We focused on BKV viremia, since BKV nephropathy with renal failure has been reported to be strongly associated with viral load in patient's blood [1]. All patients with a low viral load (< 1.9 × 10^5 ^copies/ml) had a stable renal function without treatment [1]. Thus, our results with low viral load (≤4.4 × 10^2 ^copies/ml) and only one BK positive patient revealing AKI stage 2, are in agreement with these data [1].

In immunosuppressed patients, onset of viruria and viremia occurred 40.5 days (range: 0-415 days) and 60 days (range: 18-276 days) post renal transplantation [15]. Median onset of BKV replication in urine by detecting decoy cells was estimated at 16 weeks (range: 2-69 weeks), of viral DNA in blood at 23 weeks (range: 4-73 weeks), and of BK polyoma virus associated nephropathy (PyVAN) at 28 weeks (range: 8-86 weeks) following renal transplantation [16]. Thus, only patient 5 (Table 2) was in the range, BKV nephropathy might have been detected, underlying the reported range of 56 up to 602 days [16]. Thus, our observation period may have been too short to detect BK nephropathy.

In the present study, diagnosis of AKI was based on current clinical definitions [10], which do not reflect the underlying pathogenetic mechanisms of BK-PyVAN, diagnosed by renal biopsy and histologically on tubular-interstitial nephropathy [17].

In renal transplant recipients, a high BK viral load in plasma (> 10000 copies/ml) for more than three weeks suggests that BK-PyVAN is highly likely, i.e. "presumptive BK-PyVAN" [18]. In our four patients with BK viremia, viral load was low and did not exceed three weeks, making BK-PyVAN very unlikely. Decoy cells and viral DNA in urine are used for screening purposes and response to therapeutic interventions for BK-PyVAN. Due to low viral load in blood in our patients, these screening methods might have detected more cases of BK reactivation, however, with even minor relevance for the clinician since large nucleus decoy cells do not necessarily point to the development of BK-PyVAN.

Studies in non-renal solid organ transplant recipients have shown variable prevalence of BK viruria and scarcely BK viremia during the first post-transplant year and in allograft recipients with deteriorating renal function, and presence of BKV in urine or blood was not associated with worse renal function [1,19-24]. Thus, a second, organ-specific hit, such as donor-recipient human leukocyte antigen mismatch, appeared to be necessary for the final development of BK viremia and/or nephropathy [24]. A missing second hit and an adequate humoral immune response [25] in our four BK positive patients may have contributed to avoidance of high level BK viremia and presumptive or manifest BK-PyVAN.

## Conclusions

In conclusion, BKV viremia in critically ill surgical haemorrhagic and septic shock patients appears to be rare and associated with low BK viremia, only. Thus, it is very unlikely that BKV viremia results in nephropathy later on in these patients.

## Competing interests

The authors declare that they have no competing interests.

## Authors' contributions

MN, BW, EMS and MW participated in study conception, study design, data analysis, interpretation and drafting of the manuscript. MN, MH and MW participated in collection of blood samples and data acquisition. MN and BW performed the detection of BKV DNA via real-time polymerase chain reaction (rt-PCR). All authors read and approved the final manuscript.
